# Histocompatibility in *Botryllus schlosseri* and the origins of adaptive immunity

**DOI:** 10.1007/s00251-025-01379-7

**Published:** 2025-05-10

**Authors:** Anthony W. De Tomaso, Henry Rodriguez-Valbuena

**Affiliations:** Department of Molecular, Cellular and Developmental Biology, University of California, Santa Barbara, CA 93106 USA

**Keywords:** Allorecognition, T-cells, Natural killer cells, Thymic selection, NK education

## Abstract

The basal chordate, *Botryllus schlosseri*, undergoes a natural transplantation reaction that is controlled by a single, highly polymorphic locus called the *fuhc*. The fuhc is one of the most polymorphic loci ever described, with most populations having hundreds of alleles, and up to a thousand found worldwide. Two individuals are compatible if they share one or both alleles, while those with no shared alleles are incompatible; thus, *Botryllus* uses a missing-self recognition strategy to discriminate between up to a thousand histocompatibility ligands. Remarkably, this discriminatory capability, which rivals that of vertebrate adaptive immunity, is carried out by germline-encoded receptors; thus, the mechanisms that establish and maintain this remarkable specificity are not understood. Multiple complete haplotypes of the fuhc locus have recently been sequenced, and at least seven genes with characteristics that suggest a role in allorecognition have been identified, including ligands, receptors, and intracellular proteins that likely organize and tune signal transduction complexes. This includes a new receptor family called the *fester co-receptors* (*FcoRs*) that encode ITIM and hemITAM domains, linking allorecognition in *Botryllus* to canonical immune transduction pathways. This review will summarize our current understanding and working hypotheses on the cellular and molecular mechanisms that control this innate, highly polymorphic allorecognition response, and how those may have been co-opted during the evolution of adaptive immunity.

## Introduction

Highly polymorphic allorecognition systems are found throughout the metazoan phyla, from sponges to humans (Rosengarten and Nicotra 2011; Grice and Degnan 2015; Grice et al. 2017). While allorecognition responses in vertebrates are well understood, the mechanisms underlying invertebrate allorecognition are a complete mystery. First, in the three major models of invertebrate allorecognition (sponges, cnidarians, and ascidians), there is no conservation of the candidate allorecognition proteins identified, nor are they related to any mammalian immune proteins (Grice and Degnan 2015). The universal presence of allorecognition suggests that a common selective pressure exists that would require a multicellular organism to be able to discriminate self from non-self. Yet the histocompatibility systems are not conserved, and have evolved via convergent evolution. However, they all share three characteristics. First, they show extraordinary specificity, and can discriminate between hundreds and up to a thousand histocompatibility types. Second, while recombination and mutation are the basis of discriminatory ability in the adaptive immune system, invertebrates do not have the genes (e.g., RAG or AID) that promote somatic diversification, nor are there any data suggesting that alternative mechanisms exist or are required for discrimination in these species. Third, they all share a common genomic organization: candidate allorecognition genes are multigene families encoded in haplotypes. In both cnidarians and ascidians, these are polymorphic haplotypes with gene content variation, and include both putative activating and inhibitory members (Huene et al. 2022; Rodriguez-Valbuena et al. 2024, 2022).

The structural basis of this recognition specificity and early appearance in evolution is fascinating. The ability of a T-cell to discriminate between a single amino acid substitution in a pMHC complex is only found in adaptive immunity, and based on the combination of somatic recombination and thymic selection. These processes randomly create then discover only those TCRs with the correct structural chemistry at the TCR/pMHC interface that make them competent to bind differentially (Sibener et al. 2018). And while there are innate allorecognition systems in the vertebrates (e.g., KIRs/Ly49 s and classical MHC I), the invertebrate allorecognition systems have orders of magnitude more discriminatory ability. Thus, from a functional standpoint, allorecognition in invertebrates is more reminiscent to adaptive immunity, yet novel systems have evolved independently, repeatedly, and rapidly throughout metazoan evolution, without the benefit of somatic recombination or a thymus.

As allorecognition ligands and receptors are unique to each model, we hypothesize that convergent evolution of polymorphic discrimination systems throughout the animal kingdom is founded on conserved intracellular processes that interpret binding information, and are likely the precursors to thymic education in T-cells, and the rheostat model of education in NK cells, which have also been referred to as quality control processes (Boehm 2006, 2011; De Tomaso 2009). Both thymic and NK education are cell autonomous processes which take receptor diversity, created either by recombination (T-cells) or stochastic expression of activating and inhibitory receptors (NK cells), and create specificity (Brodin et al. 2009; Joncker et al. 2009; Ashby and Hogquist 2024). These education processes set activation thresholds which allow each cell to be tolerant, but able to respond to changes in their cell surface ligands and are the foundation on which mammalian polymorphic discrimination is based. If these quality control processes appeared early in evolution, they could help explain the “big bang” appearance of two independent adaptive immune systems occurred during early vertebrate evolution—one in the jawless fish and one in the jawed fish (discussed below (Flajnik 2014)). This could also explain the even more rapid convergent evolution of NK receptors in mouse and human, whereby equivalent functions (binding to classical MHC I) are carried out by completely divergent receptors—Ly49 and KIRs, respectively (Gumperz and Parham 1995).

Conservation of intracellular decision-making processes would allow immune receptors to maintain specificity with their rapidly evolving, highly polymorphic ligands. In support of this hypothesis, we have recently found a new Ig-superfamily receptor family in the fuhc locus that encodes canonical ITIM and hemITAM motifs, linking allorecognition in *Botryllus* to canonical mammalian signal transduction pathways (discussed below; Rodriguez-Valbuena et al. 2024). These motifs were also found on candidate allorecognition genes in the cnidarian, *Hydractinia* allorecognition (*alr*) locus (Huene et al. 2022, Rodriguez-Valbuena et al. 2022). This means that allorecognition systems from cnidarians to mammals (a divergence time of ca. 700 million years), each of which utilize unrelated receptors, all converge on the same signal transduction pathways. Thus, the mechanisms which utilize these pathways to translate binding to outcome—and hence specificity—are also likely conserved. Our long-range goal is to understand how this occurs.

**Fig. 1:**
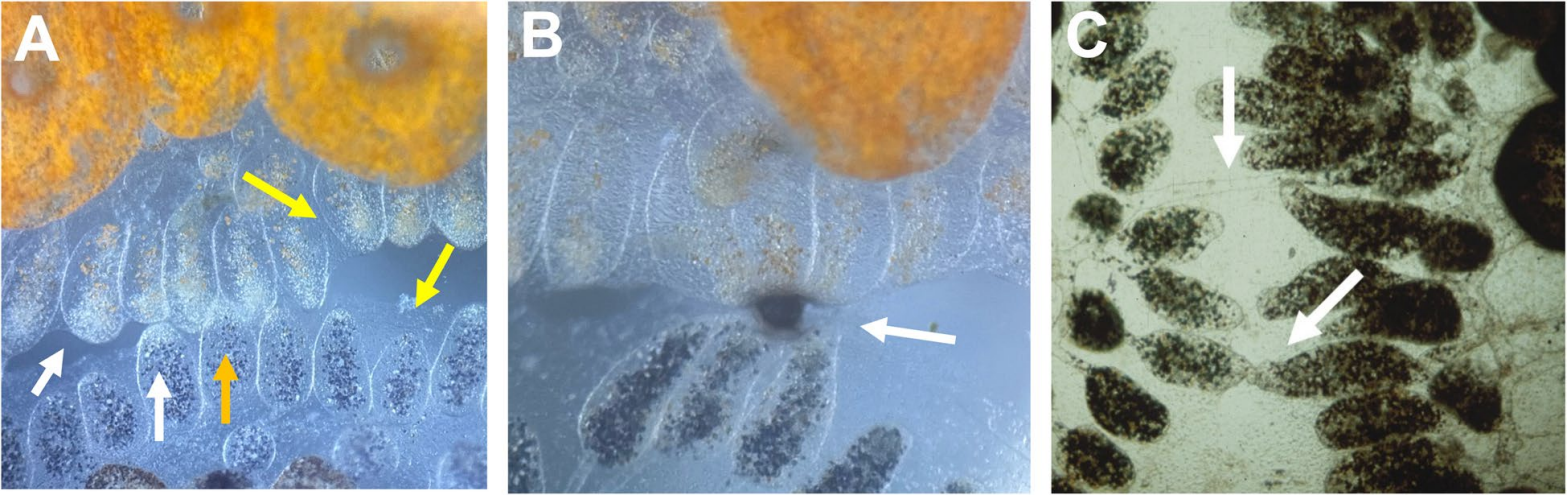
Allorecognition in *Botryllus schlosseri*. When two individuals grow into contact, the first tissues to touch are the ampullae. Panel **A** shows the initial contact between a blue (bottom) and orange (top) colony. This induces migration of a blood cell type called a morula cell to the tips of the ampullae that have come into contact. A concentration of these opaque morula cells can be seen in the ampullae (white arrow) that are not seen in ampullae distal from the contact zone (yellow arrows). The orange arrow points to an ampullae from the blue colony that has formed an immune synapse with two ampullae from the orange colony. Morula inflammation occurs during the beginning of both fusion and rejection response. **B** If individuals do not share one or both fuhc alleles, the ampullae become leaky, and the morula cells discharge, releasing the precursors of the prophenoloxidase pathway, resulting in the local deposition of melanin, forming a structure called a *point of rejection* (POR, white arrow). **C** If two individuals are compatible, the ampullae fuse together, forming a parabiosis (white arrows). The ampullae then transdifferentiate into a vessel

An excellent model of invertebrate allorecognition is the basal chordate, *Botryllus schlosseri*. Allorecognition in *Botryllus* occurs when two individuals grow into each other, and terminal projections of the vasculature, called ampullae, come into contact (Fig. 1A). This interaction results in one of two outcomes. Either the two ampullae fuse together, uniting the circulation of the two individuals, or a rejection response occurs, which is an inflammatory reaction that blocks vascular fusion (Fig. 1B, C). The decision to fuse or reject is controlled by a single, highly polymorphic genetic locus, called the fuhc (for fusion/histocompatibility Oka and Watanabe 1957; Sabbadin 1962; Scofield et al. 1982b). Two colonies fuse if they share one or both fuhc alleles, and reject if they share neither allele. The rules of fusibility demonstrate that compatibility is due to the recognition of a single self-allele on the other genotype, and there is no difference in any aspect of the fusion reaction if the two individuals share one or both alleles (Scofield et al. 1982b). The polymorphism of the fuhc locus is extremely high—most populations have > 300 alleles, and there are likely over 1000 alleles worldwide (Grosberg and Quinn 1986; Rinkevich et al. 1995; Nydam et al. 2013c); thus, the recognition system can pinpoint a self-allele from a multitude of competing specificities. In summary, *Botryllus* uses a missing-self recognition strategy to discriminate between up to a thousand histocompatibility ligands.

Functionally, the allorecognition reaction is only initiated when two *B. schlosseri* individuals come into contact, as ampullae do not reject other organisms or physical objects when they touch (Fig. 1). Thus, allorecognition consists of two separate events; one is species-specific and initiates the response, while the other is individual-specific, determining the fusion or rejection phenotype. Our findings are consistent with these observations—fusion and rejection are due to the integration of two independent inputs, although the exact mechanisms are not well understood (discussed below (Nyholm et al. 2006; McKitrick et al. 2011)). In addition, allelic discrimination is spatially segregated and takes place at the contact zone between ampullae, as a colony can simultaneously fuse to a compatible individual on one side, and reject on another (McKitrick et al 2011). Finally, while the rules of histocompatibility are that individuals that share one or both fuhc alleles are compatible and fuse, it is worth emphasizing that every ampulla in a fuhc heterozygote can recognize both fuhc alleles—an fuhc^a/b^ individual can simultaneously fuse with a fuhc^a/d^ colony on one side, and a fuhc^b/e^ colony on the other. Thus, a *Botryllus* individual can make two independent, highly specific allorecognition receptors, begging the question as to why the rules are that only a single match is required for fusion (De Tomaso 2006).

The life history characteristics of *B. schlosseri* also allow us to study the other fascinating question in histocompatibility: why it exists in the first place. Allorecognition in *Botryllus* mediates the natural transplantation of stem cells (Sabbadin and Zaniolo 1979). Following fusion of two compatible individuals, mobile germline stem cells (GSCs) migrate via the parabiotic linkage from one genotype to the other. When GSCs from two individuals co-exist in a single body, they can both contribute to germline development; thus, an individual can be a single somatic genotype, but be transmitting the gametes from another individual. This germline parasitism is under genetic control, with winners and losers found in natural populations, and given that from an evolutionary perspective loss of the germline is tantamount to death, this interaction is a strong selective pressure to maintain individual integrity (Stoner et al. 1999; Fentress and De Tomaso 2023), and is responsible for creating and maintaining the extraordinary diversity of the fuhc-based allorecognition system. Competition between cell lineages is likely the driving force for allorecognition throughout the metazoa (Buss 1982).

**Fig. 2:**
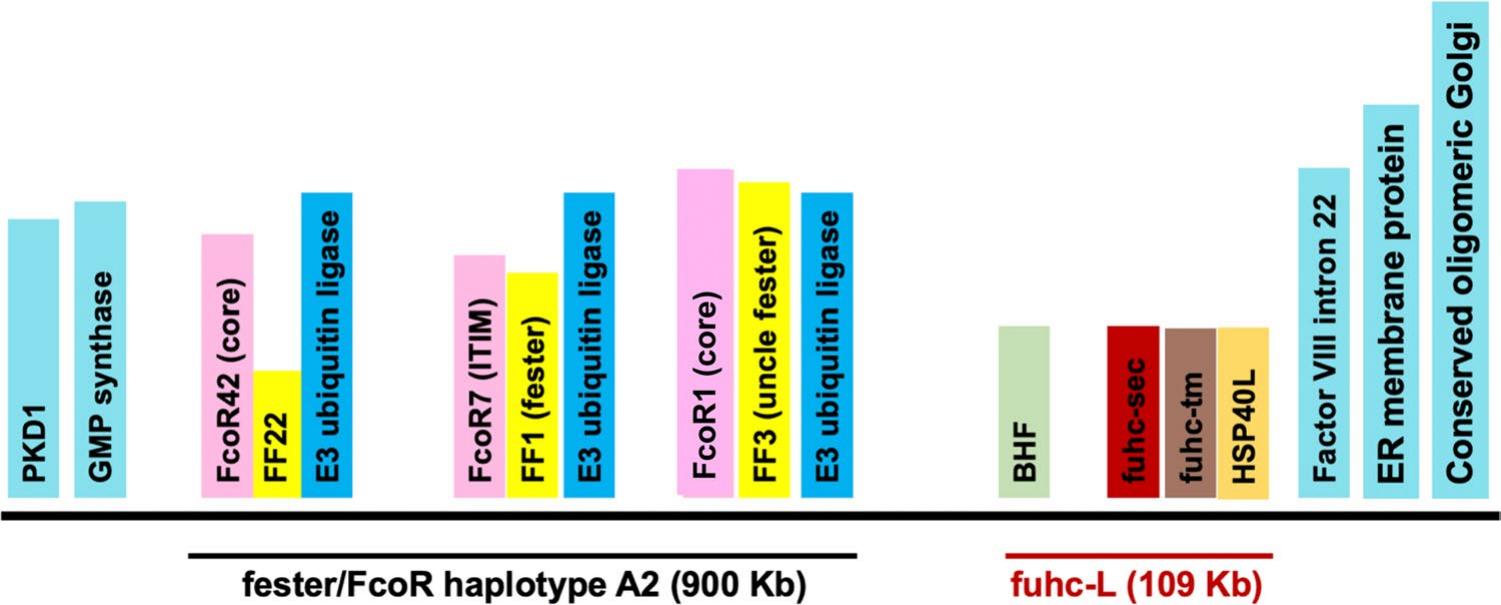
Genomic organization of the fuhc locus. An illustration of the fuhc A2 haplotype as described in the text. Conserved framework genes are shown in light blue, and these genes are conserved and demarcate the fuhc locus in all botryllid ascidians. The fuhc-L (BHF to HSP40L) is shown in red and in this haplotype spans a distance of 109 Kb. The distance between BHF and *uncle fester* (FF3) is ca. 150 Kb, and this haplotype contains three fester family (FF)/fester co-receptor (FcoR) pairs, each of which is encoded next to a HECT family E3-ubiquitin ligase gene (*HE3L*). The fester/FcoR region is highly diverse between haplotypes, and gene content variation is of pairs of FF/FcoR genes (Rodriguez-Valbuena et al. 2024). The signaling domain of each FcoR is show; the core motif is the sequence (Y-x-x-I/L/V). Drawing not to scale. FF fester family, FcoR fester co-receptor

Besides the phylogenetic relationship to vertebrates, one advantage of *B. schlosseri* as a model is the simple genetics that control fusibility. Analysis of over 100 independent crosses done in multiple populations from independent groups throughout the world all clearly demonstrates that the fuhc is a single locus: fusibility segregates in a simple Mendelian fashion, even in wild-type crosses—there are no modifying loci or other genetic processes at play (Oka and Watanabe 1957; Sabbadin 1962; Scofield et al. 1982a, b). In contrast, both sponges and cnidarians appear to have more complex genetic systems (Grice et al. 2017; Rodriguez-Valbuena et al. 2022). More importantly, the fuhc locus is turning out to be remarkably informative. Encoded within the ca. 800 Kb locus are ligands, receptors, and intracellular molecules that likely play a role in specificity (Fig. 2). Polymorphism is a key characteristic of proteins involved in allorecognition, and is found in seven proteins in the locus, named:*fester*, the *fester co-receptor* (*FcoR*), a HECT family E3-ubiqutin ligase (*HE3L*), *Botryllus histocompatibility factor* (*BHF*), *fuhc-sec*, *fuhc-tm*, and *HSP40L*. Four of these genes (*BHF/fuhc-sec/fuhc-tm/HSP40L*) are encoded in an ca. 110 Kb region of the genome (Fig. 2; De Tomaso et al. 2005; Voskoboynik et al. 2013; Rodriguez-Valbuena et al. 2024; Thier et al. 2024)). Thus far, polymorphism of all four genes segregates with fusibility outcomes, suggesting that one or a combination of the genes is the ligand or required for specificity; thus, we are calling this region the fuhc-L (Fig. 2). The genomic organization of the fuhc-L is highly conserved both between *B. schlosseri* individuals, and also throughout the botryllid ascidians, which all have equivalent single locus allorecognition systems (Saito et al. 1994; Rodriguez Valbuena, in preparation). In contrast, the region of the fuhc encoding the allorecognition receptors *fester*, *FcoR*, and *HE3L* is highly polymorphic—the three genes are encoded in diverse clustered haplotypes that are rapidly evolving, with gene content variation that differs greatly between *B. schlosseri* individuals, and also at the species level. The fuhc region is bounded by conserved housekeeping genes (Fig. 2), and these genes are conserved in the fuhc of all related species examined thus far, and similar to the housekeeping genes of the Class III region of the vertebrate MHC, are conserved even in distantly related species that do not encode any fuhc effector genes (Rodriguez-Valbuena, in preparation; Rodriguez-Valbuena et al. 2024).

In summary, the fuhc locus of *B. schlosseri* appears to be analogous to the concept of the ancestral, ur-MHC supergene: both ligands and receptors are encoded side by side (Flajnik and Kasahara 2010; Flajnik 2014), although as described below we have recently uncovered another level of complexity in the effector arm. Importantly, this locus also encodes three surprisingly polymorphic cytoplasmic proteins that could help us understand the basis of specificity: *BHF*, *HSP40L*, and *HE3L*. As discussed below, our data suggest that these proteins could be involved in establishing and/or maintaining specificity (Taketa et al 2015).

## Positional cloning of the fuhc locus—the fuhc ligand (fuhc-L)

*Botryllus schlosseri* colonies can be reared and crossed entirely in the laboratory, and partially inbred lines of *B. schlosseri* with known fusibility types have been created (De Tomaso et al. 1998). Using these resources, the fuhc locus was genetically and physically mapped, and candidate genes from the genomic sequence were isolated and studied (De Tomaso et al. 2005). The genetic map delineated the fuhc to a 1.3 cM region, and physical mapping of this region resulted in several contigs that could not be assembled, but represented over 1.0 Mb of DNA. A new genome assembly confirms this, giving a conservative estimate of genetic to physical distance of 1 cM = 1 Mbp, as well as providing the complete genome sequence of two fuhc haplotypes from an individual from the Mediterranean coast of France (Rodriguez-Valbuena et al. 2024; Thier et al. 2024). Genes within the physically mapped fuhc were predicted computationally, and candidates for further studies were chosen based on their predicted properties, e.g., known protein domains (e.g., Ig or LRRs), predicted architecture for a histocompatibility ligand (signal sequence and transmembrane domain), or anything else that looked vaguely immune-like. These were next isolated from cDNA made from two rejecting genotypes and tested for polymorphism, and if so, were studied further.

### Fuhc-sec and fuhc-tm

**Fig. 3:**
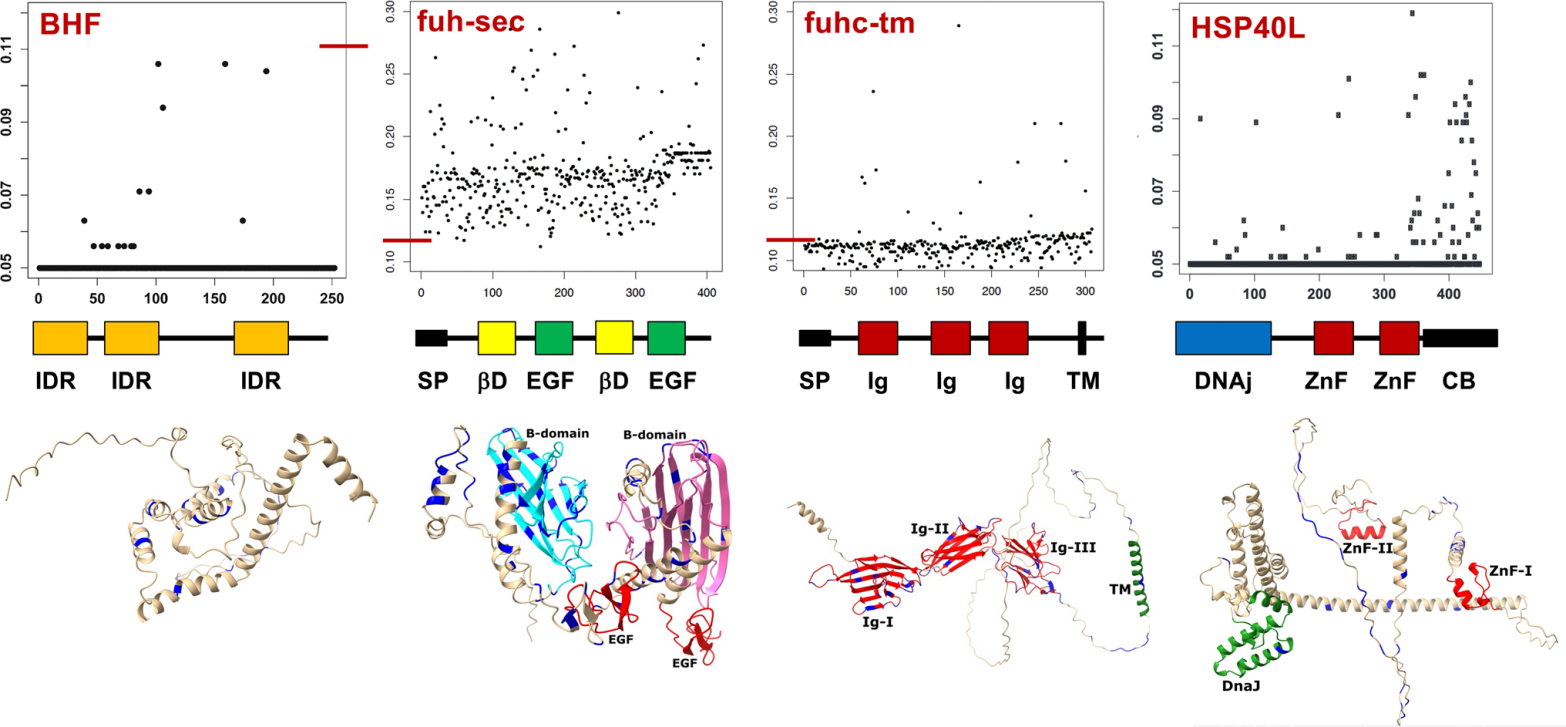
Predicted structure and distribution of polymorphism in the four fuhc-L genes. **Top**: DIVAA analysis of the distribution of polymorphism among 20 + allotypes of the four fuhc-L proteins is shown. Each amino acid is a dot, and plotted using the DIVAA metric, whereby a value of 0.05 means complete conservation of the amino acid at that position among all allotypes, while a value of 1.0 means that every allotype has an equal probability of encoding any of the 20 amino acids at that site (Rodi et al. 2004). Note change in scale for BHF and HSP40L vs fuhc-sec and fuhc-tm (red lines). The black line at 0.05 for BHF and HSP40L is composed of hundreds of black dots, as the vast majority of each protein is completely conserved. Only polymorphic residues in the ectodomain are shown for fuhc-sec and fuhc-tm. No hypervariable regions found in any gene; however, the majority of substitutions of the HSP40L allotypes are concentrated in the client binding domain at the COOH tail of the protein, which interacts with target proteins (Kampinga and Craig 2010). **Middle**: A 2D model of protein domain structure for each locus is shown (not to scale). Domains are as follows: IDR, intrinsically disordered region; SP, signal peptide; EGF, epidermal growth factor; Ig, immunoglobulin; TM, transmembrane, Zn-F, zinc finger domain; CB HSP 40, client binding domain. **Bottom**: Alphafold models of each gene are shown. Polymorphic residues with ≤ 80% (HSP40L) or ≤ 89% (fuhc-Tm and fuhc-Sec) identity in alignments of the HSP40L, fuhc-tm, and fuhc-sec proteins are highlighted in blue. The proteins are oriented with the N-terminal regions to the left side

One gene had most of the characteristics expected for a histocompatibility ligand which we named the candidate fuhc (*cfuhc*): it was a highly polymorphic transmembrane protein, the polymorphisms segregated with fusibility in wild-type pairings, the protein was expressed in the blood and ampullae, and it was an immunoglobulin superfamily member (De Tomaso et al. 2005). In our first assessment, we found that this was a single gene that could be alternatively spliced into a secreted form, and a transmembrane form. Later we found that candidate fuhc was not a single, alternatively spliced gene, but actually two genes encoded side by side, a secreted protein (*fuhc-sec*), and a transmembrane protein (*fuhc-tm*) separated by 227 bp (Figs. 2 and 3) (Nydam et al. 2013a, b, c). *fuhc-sec* is a ca. 550 residue protein with a signal sequence, Ig domain and two EGF domains, and significantly more polymorphic than any other gene in the fuhc locus, with 21 codons showing evidence of balancing selection (Nydam et al., 2011). The distribution of polymorphism is interesting—each allele differs by ca. 25 residues spread throughout the protein, and there are no hypervariable or other regions that provide insight into allelic discrimination (Fig. 3). Importantly, there are no transmembrane or other predicted domains that would target the protein to the cell surface, and it is secreted when expressed in mammalian cells (Nydam et al. 2013b). Nevertheless, both functional and molecular evolutionary studies suggest that fuhc-sec is the ligand (discussed below).

*fuhc-tm* is a ca. 535 residue type I transmembrane protein with a signal sequence, three tandem Ig domains, a tm domain, and short intracellular tail (Fig. 3). Each allele of fuhc-tm differs by about 10–15 amino acids which are also scattered throughout the ectodomain, and only 5 codons show evidence of balancing selection (Fig. 3; Nydam et al., 2011). *fuhc-tm* is targeted to the plasma membrane when expressed in mammalian cells (Nydam et al. 2013b), and the tight linkage and polymorphism of the two proteins might indicate that the fuhc ligand is a *fuhc-sec/fuhc-tm* heterodimer. However, this would require exclusive assembly of the genes from each haplotype in a fuhc heterozygous individual. In turn, this would necessitate co-evolution of the two alleles to maintain specificity, which seems unlikely given the number of histocompatibility types in natural populations. In addition, while both genes are co-expressed in the ampullae, scRNA seq of circulatory cells has revealed that the two genes are expressed on different populations—*fuhc-sec* is on a population of blood cells, which was also seen in an independent study (Rosental et al. 2018). However, *fuhc-tm* is found on a subpopulation of germline stem cells (in preparation). This is interesting given that there are no promoter or enhancer elements in the 227 bp region that separates the two genes. Co-expression studies demonstrate that *fuhc-tm* and *fuhc-sec* can form a heterodimer at the cell surface (Nydam et al 2013b); however, other role(s) of *fuhc-tm*, and more importantly, the polymorphism of the protein, are not understood at this time.

### HSP40L

These studies also identified a polymorphic HSP40/chaperone protein encoded 8 Kb away from the *fuhc-tm* locus and also expressed in the ampullae (Fig. 2 and 3 (Nydam et al. 2013a). *HSP40L* is closely related to the DNAjC21 protein. It is nearly the exact same length and has identical protein domain architecture, consisting of an NH terminal HSP40 domain, two zinc fingers interspersed with coil/coil domains, and a COOH tail “client binding” domain which interacts with target proteins (Kampinga and Craig 2010). The fuhc-encoded gene is polymorphic, and these polymorphisms are concentrated in the client binding region at the COOH tail of the protein, and multiple codons show evidence of selection (Fig. 3). When expressed in mammalian cells, HSP40L was found in the cytoplasm (Taketa et al. 2015). To our knowledge, there is no other example of a polymorphic DNAj family member, and these characteristics suggest a role in assembling allele-specific protein complexes, and/or signal transduction (Lang et al. 2022).

### Botryllus histocompatibility factor (BHF)

An unbiased bioinformatics screen designed to identify all the polymorphic genes in transcriptomes from multiple fuhc-defined genotypes and assess their correlation with fusibility identified a gene called *Botryllus histocompatibility factor* (*BHF*) (Voskoboynik et al. 2013). Polymorphisms of *BHF* correlated with fusibility outcomes, and the gene was found to be encoded in the fuhc locus, 67 Kb away from *fuhc-sec* (Fig. 2), confirming previous genetic studies. *BHF* is a protein with multiple intrinsically disordered regions (IDR) that is also polymorphic—and similar to HSP40L, to our knowledge, this has not been previously described. That said, it is not clear what role polymorphism would play in an IDR, although newer modeling techniques suggest that most of the polymorphisms are found in α-helices between the IDR regions (Fig. 3).

Knockdown of BHF blocked both fusion and rejection responses, resulting in a “no-reaction” phenotype, equivalent to the knockdown phenotypes of the inhibitory receptor, *fester* (discussed below, Nyholm, et al. 2006). While it was initially suggested that *BHF* is a histocompatibility ligand, a subsequent study found that *BHF* is neither highly polymorphic, nor is there strong evidence of positive or balancing selection on the protein (Fig. 3; Taketa et al. 2015), with the caveat described above. In addition, multiple tagged versions of *BHF* were expressed in mammalian cells and the protein was targeted directly underneath the plasma membrane. Interestingly, *BHF* also remained associated with the plasma membrane following subcellular fractionation, and using a series of truncation and point mutation constructs, we found that three cysteines in the first 50 amino acids had a significant role in *BHF* localization, likely due to palmitoylation of those residues (Taketa et al. 2015). IDPs can play a critical role in signal transduction, including signal tuning and integration, often based on their role in phase transitions or the ability to act as promiscuous signaling hubs (Bondos et al. 2022). The combination of polymorphism and loss-of-function phenotypes suggests that BHF is involved in assembling and maintaining membrane protein complexes that include *fester*, and these complexes are responsible for signal transduction and specificity (discussed below). This is consistent with the targeting of BHF to the intracellular side of the plasma membrane, as we hypothesize that clustering and/or spatial coordination of proximal signaling is critical for polymorphic discrimination. BHF may provide a novel tool to dissect the molecular interactions that underlie polymorphic discrimination in *B. schlosseri*.

## The fuhc effector system

### The fester family

The first highly polymorphic gene identified in the fuhc locus was *fester*, which was a 350 amino acid type I transmembrane protein with a signal sequence, an extracellular Ig domain, a Sushi domain, followed by three transmembrane domains, and a short intracellular tail (Nyholm et al. 2006; Rodriguez-Valbuena et al. 2024). *Fester* is both polymorphic, there are ca. 100 allotypes in the US, and polygenic, as two haplotypes were found in our lab-reared strains, one with a single fester locus, and another with two loci. However, *fester* is not as polymorphic as *fuhc-sec*, nor did the polymorphisms correlate exactly with fusibility outcomes. In addition, it appeared that multiple exons of *fester* can be alternatively spliced, with each individual analyzed expressing a full-length form of fester, along with a unique repertoire of alternative splice variants. Importantly, genotype-specific alternative splice repertoires were maintained for the life of the individual—the ampullae can be ablated and will regenerate with no loss of specificity, and the fester splice repertoire was maintained, even following multiple ablation/regeneration cycles (Nyholm, et al. 2006). Together, these genotype-specific characteristics suggested that *fester* was an allorecognition receptor responsible for discrimination of fuhc ligands, and we speculated that avidity from binding of different splice variants could be responsible for specificity. As described below, this is turning out to be a much more complex story (Rodriguez-Valbuena et al. 2024).

*Fester* is expressed on the ampullae as well a subset of blood cells, and its role in allorecognition was assessed using two methods—monoclonal antibody (mAb) interference and siRNA knockdown. When a monoclonal antibody raised to an allele (*fester*^*A*^) carried in our lab-reared lines was injected into individuals in a compatible pairing—both *fester*^*A/A*^ homozygotes, there was no effect, and fusion occurred normally. However, when injected into individuals that were incompatible, but both expressed the *fester*^*A*^ allele, a rejection could be turned into a fusion, suggesting that the antibody was stimulating *fester*, which could override the rejection response. This result was allele-specific, but more importantly we found that one of the two individuals in the preceding experiment had to be a *fester*^*A/A*^ homozygote—if the antibody was injected into two incompatible *fester*^*A/B*^ heterozygotes, the rejection response was not blocked. This indicated a dose-dependent response to mAb binding was required to override the rejection response, and that ampullae from both individuals had to be stimulated for fusion to occur. In contrast, siRNA knockdown of *fester* gave an unexpected phenotype. Given the mAb-blocking results, we expected that knockdown of *fester* would cause compatible pairings to reject, as there would be no recognition of the fuhc ligand. However, we found that knockdown of fester blocked both fusion and rejection responses—when ampullae of either compatible or incompatible pairings came into contact, there was no reaction, equivalent to that seen for BHF (discussed above). As the siRNA was complementary to non-spliced exons of fester and should have ablated all splice variants, we hypothesized that while the spliced repertoire provided specificity, either the full-length form common to all individuals or a subset of splice variants could be partitioned into a receptor controlling rejection and another controlling fusion.

Subsequently, we identified another genomic locus that appeared to be a duplication of the region encoding *fester* exons 6–11, but with no homology to the 5′ end of the gene, and discovered it encoded a related protein, *uncle fester*. The NH2 terminal half of *uncle fester* had no DNA or amino acid homology with *fester*, but an equivalent architecture, including a signal sequence, Ig and sushi domain, followed by three TM domains, and an intracellular tail. In contrast to *fester*, *uncle fester* was monomorphic, and we only identified two alleles that differed by a single amino acid in populations from the USA and Europe. In addition, *uncle fester* did not show extensive alternative splicing; however, we found that there were genotype-specific expression levels that differed by over tenfold between individuals, and were stable over time (Taketa et al. 2024).

*Uncle fester* was also expressed in the ampullae and a subset of blood cells, and we carried out equivalent functional studies as described above. First, siRNA knockdown of *uncle fester* in incompatible pairings resulted in a no-reaction phenotype, blocking the rejection response, equivalent to *fester*. In contrast, knockdown of *uncle fester* in compatible pairings had no effect, and the ampullae fused normally, indicating that *uncle fester* is solely involved in the rejection pathway, and that fusion and rejection are not mutually exclusive. In addition, these studies also revealed that uncle fester expression level correlated with the rejection phenotype. For example, partial knockdowns resulted in a slower, less intense rejection response vs control pairings, and genotypes with higher expression would have robust rejection responses (McKitrick et al. 2011; Taketa et al. 2024). We also carried out mAb interference experiments and found that the presence of an anti-*uncle fester* mAb had no effect on incompatible pairings, which rejected normally. In contrast, in compatible colonies the presence of the antibody would override a fusion in process, resulting in a rejection. Finally, by conjugating the mAb to magnetic beads we could manipulate an individual ampullae, which initiated a rejection response, but had no effect on adjacent ampullae, consistent with the spatial segregation of the reaction, described above. In summary, *uncle fester* is necessary and sufficient to activate the rejection response.

Together, these studies revealed that allorecognition outcomes are not mutually exclusive, rather a quantitative response due to the integration of signals from two independent pathways, each of which is tunable. When juxtaposed ampullae come into contact and form a synapse, a rejection response is initiated at the immune synapse via a receptor that includes *uncle fester* (Fig. 1A). This recruits a blood cell type called a morula cell to the tips of the ampullae in contact, and recent scRNA seq data suggests this inflammatory signal is mediated by IL-17 (in preparation). If no other signal is received, the ampullae lose barrier function, and the morula cells leak into the periphery and lyse, discharging the precursors of the prophenoloxidase pathway, which form melanin scars in situ, called points of rejection (POR; Fig. 1B). This reaction also produces ROS that can kill the nearby the ampulla (Ballarin et al. 1998, 2002). As described below, there are a range of rejection responses that can be classified by the number, size, and time of formation of the POR, as well as the integrity of the interacting ampullae. However, if a self fuhc allele is recognized by fester, this overrides the rejection response and triggers fusion of the ampullae, which transdifferentiate into a vessel (Fig. 1C).

Functionally, this is equivalent to responses by mammalian NK cells, which integrate signals from activating and inhibitory receptors via integration of ITIM and ITAM signaling; however, neither *fester* nor *uncle fester* encodes any known signaling domains. Nonetheless, we had already found that proteins encoding cytoplasmic ITIM and ITAM domains—as well as nearly all associated signal transduction molecules used in mammals—were present in ascidian and other invertebrate genomes (Azumi et al. 2003; Nicotra 2019), and are expressed in the ampullae (see below), so hypothesized that signaling was transmitted via promiscuous adaptor molecules encoded activating (e.g., a DAP12 homolog) and inhibitory motifs. In addition, while the knockdown phenotypes were robust and consistent in different genotypes, it was not clear if other receptors contributed to specificity. As described below, we have now found the signal transduction partner, which has revealed another level of complexity to the effector system.

### HECT family E3-ubuiqutin ligase (HE3L)

Finally, in our initial studies, two haplotypes from our mapping populations were physically mapped (Nyholm et al. 2006). Haplotype A had a single *fester* and single *uncle fester* locus, while haplotype B had two *fester* loci and one *uncle fester* locus. We found that a unique *HE3L* locus was encoded next to each *fester* and *uncle fester* locus in both haplotypes. The amino acid sequences were ca. 95% identical, revealing another unexpectedly polymorphic protein encoded in the fuhc locus (Rodriguez-Valbuena et al. 2024; Nath and Isakov 2024). Multiple E3-ligases, such as c-cbl, cbl-b, and NEDD4, play critical roles in tuning of both T-cell and NK signaling pathways, regulating signal strength and setting activation thresholds for both cells (O’Leary et al. 2015; Nath and Isakov 2024). For example, in naïve peripheral T-cells, ablation of cbl-b allows naïve T-cells to be activated without co-stimulation (Chiang et al. 2000), while in NK cells, c-cbl sets a threshold that can only be overcome by two independent activation signals (Kim et al. 2010). Moreover, in peripheral T-cells, the HECT family member NEDD4 positively regulates signal strength by interacting with both cbl-b and PTEN (O’Leary et al. 2015). The genomic redundancy, polymorphism, and linkage of the *HE3L* genes within the fuhc locus of *Botryllus* suggest a role in allele-specific tuning and discrimination.

### Expanded fester and the fester co-receptor (FcoR) families

We have recently found that the fester family is much more diverse than previously described, and have identified over 37 new loci. These are encoded in polymorphic haplotypes with gene content variation, and each individual expresses an average of 10 loci (Rodriguez-Valbuena et al. 2024). Similar to *fester* and *uncle fester*, the new loci share little amino acid homology but equivalent domain architecture, and we are renaming these genes the fester family—*fester* is now *FF1*, and *uncle fester* is *FF3*. We have also identified the signal transduction partners, which are another large multigene family thus far consisting of 53 loci, that we are calling the *fester co-receptors* (*FcoRs*). The FcoRs are type I TM proteins related to fester and encoded in the fuhc locus (Fig. 2). They range in size from 520 to 610 aa, and encode a signal sequence, a variable type Ig domain, sushi domain, two C2-type Ig domains, a transmembrane domain, and a long intracellular tail which can encode canonical ITIMs, hemITAMs, and a core tyrosine motif (Y-x-x-I/L/V) of unknown function (Bauer and Steinle 2017). The core motif can be found alone or in combination with the ITIM or hemITAM domains, and many loci also encode SH2 domains. Each FF family member is encoded next to and expressed with a cognate FcoR family member. For example, *fester* (FF1) is paired with *FcoR7*, while *uncle fester* (FF3) is paired with *FcoR1* (Fig. 2). There is also a link between polymorphism of each gene in a pair, and the putative functional role of the FcoR partner. For example, FcoR7 is as polymorphic as *fester*, and also encodes an ITIM domain, consistent with its role as an inhibitory receptor. By contrast, *uncle fester* (FF3) is paired with FcoR1, which is also monomorphic and encodes a tyrosine core motif domain, matching its role as an activating receptor.

These studies have also revealed several other surprising results. First, while only several haplotypes have been sequenced, the FF/FcoR are encoded as pairs in two polymorphic haplotypes—one is in the fuhc locus on chromosome 11 (Fig. 2), and another is on chromosome 5—and gene content variation in both haplotypes is of the gene *pairs*. These pairs are also co-expressed in transcriptomes from multiple individuals (*n* = 25), suggesting they assemble into specific heterodimers. To our knowledge, haplotype variation of gene pairs has never been described before, and if these genes do heterodimerize, the binding site will consist of two IgV-like domains. Finally, there is a third polymorphic haplotype found on chromosome 9 that encodes only FcoR loci.

In addition, there is only one highly polymorphic locus of both genes—FF1 and FcoR7, and these are paired. There are also two oligomorphic pairs, with four alleles discovered of each partner thus far, and a number of monomorphic pairs that were identical in > 5 individuals were also found. However, about 40% of the loci have only been identified in a single individual, so this is likely an underestimate. Nevertheless, given their role in allelic discrimination, we would have predicted that there would be more polymorphic, inhibitory gene pairs. The presence of multiple monomorphic pairs is intriguing, as the FcoR partner always encodes a hemITAM or core motif, but never an ITIM. The oligomorphic pairs are particularly interesting, as the polymorphisms in the FF partner are concentrated in the ectodomain, but the FcoR partner alleles differ in both the ectodomain and signaling motifs, and encode either an ITIM or a hemITAM. In addition, each oligomorphic FcoR locus can undergo alternative splicing that swaps one motif for the other, suggesting that the link between binding and signal output (activation or inhibition) is plastic, and required for allelic discrimination. However, the big surprise was that the *uncle fester* pair (FF3/FcoR1), which we thought was the sole activating receptor, is not ubiquitous. In summary, our previous hypotheses that allorecognition is due to the integration of activating and inhibitory pathways initiated by two receptors was oversimplified; this is a much more complicated interaction (discussed below).

Analysis of individual transcriptomes reveals that nearly all FF and FcoR genes show evidence of genotype-specific expression and alternative splicing that can differ by orders of magnitude, as we had found previously (Nyholm et al. 2006; Taketa et al. 2024). However, similar to the original results for cfuhc (described above; Nydam et al. 2013b), the extent of alternative splicing of *fester* (FF1) assessed by PCR does not match that found by either short read or long read bulk transcriptome sequencing from multiple individuals. This is intriguing, because all of the 64 splice variants of *fester* originally identified by PCR were in frame, while exons randomly mixed and analyzed computationally were not. Moreover, we had done several qPCR studies on individual splice variants that were entirely consistent, but the same variants are not found in bulk sequence data. Finally, several of these either extremely rare or non-existent splice variants were expressed in mammalian cells and targeted to the plasma membrane, indicating they fold correctly (Taketa and De Tomaso 2015). This is the second time that splice variants identified by PCR—and found at the plasma membrane when expressed ectopically—were not found in bulk transcriptome studies, and it is interesting that these are the two most highly polymorphic proteins in the fuhc locus. By contrast, this is not the case in the other 30 + genes encoded in the fuhc locus which were initially characterized using old-school RACE and PCR techniques, including *uncle fester*, which has two splice variants that were equivalent using both techniques. Finally, one consistent finding between all studies is that the vast majority of genotype-specific alternative splicing in all FF and FcoR is of the exons between the TM domain and the Ig/Sushi region, which would reposition the NH2 half of the protein relative to the plasma membrane.

### ITAM and ITIM signal transduction

As described above, ITAM and ITIM domains were identified on candidate allorecognition genes in the cnidarian, *Hydractinia*, and the genome encodes associated signal transduction molecules (Rodriguez-Valbuena et al. 2022). In *Botryllus*, all fuhc-encoded proteins, as well as a homolog of nearly every protein in the ITAM and ITIM signaling pathway used by mammalian T- and NK cells, are expressed in the *Botryllus* ampullae. This includes receptor tyrosine phosphatases with homology to CD45, Src and syk family kinases, shp-1, SHIP, VAV, PI(3)K, PLC-γ, ITK, GADS Grb2, Crk, RasGRP, PKC-θ, the MAPK pathway, CARMA1, calcineurin, calmodulin, NFAT, and NFkB. However, there are two notable exceptions: homologs of LAT and slp-76 are not found in either *Botryllus* or other ascidian genomes (Rodriguez-Valbuena et al. 2024; Azumi et al. 2003).

## Potential mechanisms of polymorphic discrimination

### Evidence that fuhc-sec is the histocompatibility (inhibitory) ligand

As described above, mAb crosslinking studies of either *fester* or *uncle fester* revealed that fusion or rejection is not mutually exclusive, but due to a balance of the two signals each of which could override the other. The role of signal integration in outcome was also demonstrated in a previous genetic study focused on understanding phenotypic differences in the rejection response, and indicates that *fuhc-sec* is the ligand (Scofield and Nagashima 1983). It has been shown that rejection responses can be binned into four distinct phenotypes (severe, moderate, mild, minimal) based on the number and size of rejection scars (Fig. 1B), as well as the timing of their appearance, which can range from 12 h (severe) to > 60 h (minimal). These rejection phenotypes are robust—independent pairings of the same incompatible genotypes result in equivalent rejection phenotypes. Genetic analysis revealed that the severity of rejection mapped back to the fuhc locus, suggesting that the strength of the rejection response could be due to differences between alleles of the self-ligand (Scofield and Nagashima 1983). The integration between rejection and fusion pathways would predict that pairings of incompatible individuals expressing closely related histocompatibility allotypes would partially recognize each other, moderately stimulating the inhibitory (fusion) pathway, in turn lowering the severity of the rejection response.

We have been using natural polymorphism to test this directly, by correlating the severity of rejection to the number and distribution of polymorphisms on candidate fuhc genes. In our original genetic mapping, a pair of individuals were identified that had a minimal rejection response, and we found that the two genotypes had closely related *fuhc-sec* alleles, but normal differences between the other candidate genes (Nydam et al. 2013b). Specifically, while the average difference between any two *fuhc-sec* alleles is ca. 25 amino acids, the two most closely related alleles in this pair differed by only 6, indicating that *fuhc-sec* is the ligand, and that the *Botryllus* allorecognition system can discriminate between two 550 aa allotypes that differ by only 1% of their protein sequence. We have now extended this study, identifying multiple genotypes with different phenotypes. This natural variation is a powerful tool to dissect allorecognition, as we can correlate any genetic or biochemical results with fusion and a range of rejection phenotypes. This is analogous to mammalian studies which used the OT-1 TCR and altered peptide ligands to probe the quantitative characteristics of positive selection (Hogquist et al. 1994) and kinetic proofreading in ligand discrimination (Voisinne et al. 2022).

### Speculations on the mechanics of polymorphic discrimination

The invertebrate allorecognition systems have discriminatory ability that is orders of magnitude higher than anything found in the innate branch of vertebrate immunity, so there is nothing obvious on which to base our predictions. However, any working hypotheses must be consistent with previous results, and importantly, explain how new specificities could evolve.

Since the *Botryllus* effector system can discriminate between up to a thousand histocompatibility types (Nydam 2020), and a heterozygous individual can make two specific receptors using germline encoded genes, it is not likely that specificity is determined based only on affinity, such as a lock and key interaction between cognate ligand/receptor pairs, or homotypic interactions, as has been suggested for *Hydractinia* (Karadge et al. 2015). First, only a single gene with characteristics expected of a histocompatibility ligand, such as high diversity between allotypes with strong evidence of natural selection, is found in the fuhc locus—*fuhc-sec* (Nydam et al. 2013c). In addition, allotypes of the polymorphic FF and FcoR genes do not correlate absolutely with fusibility, and both genes are organized and evolving by birth and death mechanisms similar to those in the mammalian NKC and LRC—exactly as would be expected for innate receptors co-evolving with a highly polymorphic ligand (Kelley et al. 2005). Furthermore, the unique complexity of the fester and FcoR genomic organization, including the presence of both linked and unlinked FF/FcoR haplotypes, gene content variation of FF/FcoR gene pairs, and a third FcoR haplotype, together make the receptors highly evolvable, and are not surprising given the required discriminatory capability of this system. These characteristics are not consistent with a simple lock and key or homotypic interaction model.

Second, the real problem with a lock and key or homotypic binding strategy is explaining how new specificities arise, which would require either complementary mutations in two genes or mutations in one gene that maintain homotypic binding. It is difficult to understand how this requirement could create the diversity seen in natural populations, as each new histocompatibility type would require ever higher specificity to develop. There are well-studied lock and key recognition systems; for example, the self-incompatibility (SI) interaction between pollen and flower that prevents inbreeding in several families of plants (Nasrallah 2005; Murase et al. 2024). However, individual populations have on the order of 10–30 SI specificities (Mable et al. 2003; Maenosono et al. 2024), not the > 200 histocompatibility types seen in *Botryllus* populations. And even in context of lower diversity, understanding how a new SI specificity could arise is not easy to grasp—the complementary mutations on the lock and key genes must occur on the exact same haplotype to be functional, and any intermediate forms would have a lower fitness and should be selected against (Charlesworth 2000). Similar arguments can be made for studies in *Hydractinia*, and it is extremely unlikely this mechanism could account for the level of diversity seen in *Botryllus*. So how might this work?

As described above, loss-of-function assays demonstrated that *fester* and *uncle fester* were necessary and sufficient for fusion and rejection responses. Knockdown of each resulted in a no-reaction phenotype, during which the ampullae touched, but there was no subsequent morula cell infiltration or downstream responses (Fig. 1A). Our recent findings that the *fester* genes are significantly more complex than originally thought indicate that the allorecognition receptors bind or signal as oligomers, and that both activating and inhibitory signaling is due to changes in cooperative binding, and each event is counted. This is no surprise; oligomerization is a requirement for signal initiation in many receptors (Duke and Graham 2009), but how would this be extended to allelic discrimination? NK cells discriminate the fate of a target cell by quantifying the expression of health and stress ligands using activating and inhibitory receptors. The differences between activating and inhibitory signals are integrated via both physical changes in activating and inhibitory receptor clustering as well as downstream signaling, and a decision to kill the target or not is made. For the former, two groups have shown that inhibitory receptor engagement blocked the formation of activating receptor microclusters, and this was consistent despite the fact that different activating and inhibitory receptors were being assessed (Abeyweera et al. 2011; Liu et al. 2012). Both studies also found that receptor microclusters accumulate at the immune synapse, and here there may be differences depending on the receptors; for example, the activating receptor CD16 accumulated at the immune synapse following ligand engagement despite inhibitory signals from the inhibitory receptor CD94/NKG2 A (Liu et al. 2012), while in a separate study, inhibitory signaling from KIR2DL1 blocked accumulation of the ligand bound activating receptor NKG2D (Endt et al. 2007). However, both reports showed that inhibitory signaling caused dephosphorylation of VAV and other downstream changes. Two important findings for our working hypotheses came from these studies. First was that activating signals could be initiated from dispersed microclusters (Abeyweera et al. 2011), and second was that inhibitory receptors can block signaling of activation receptors even if both accumulate at the synapse (Liu et al. 2012).

**Fig. 4:**
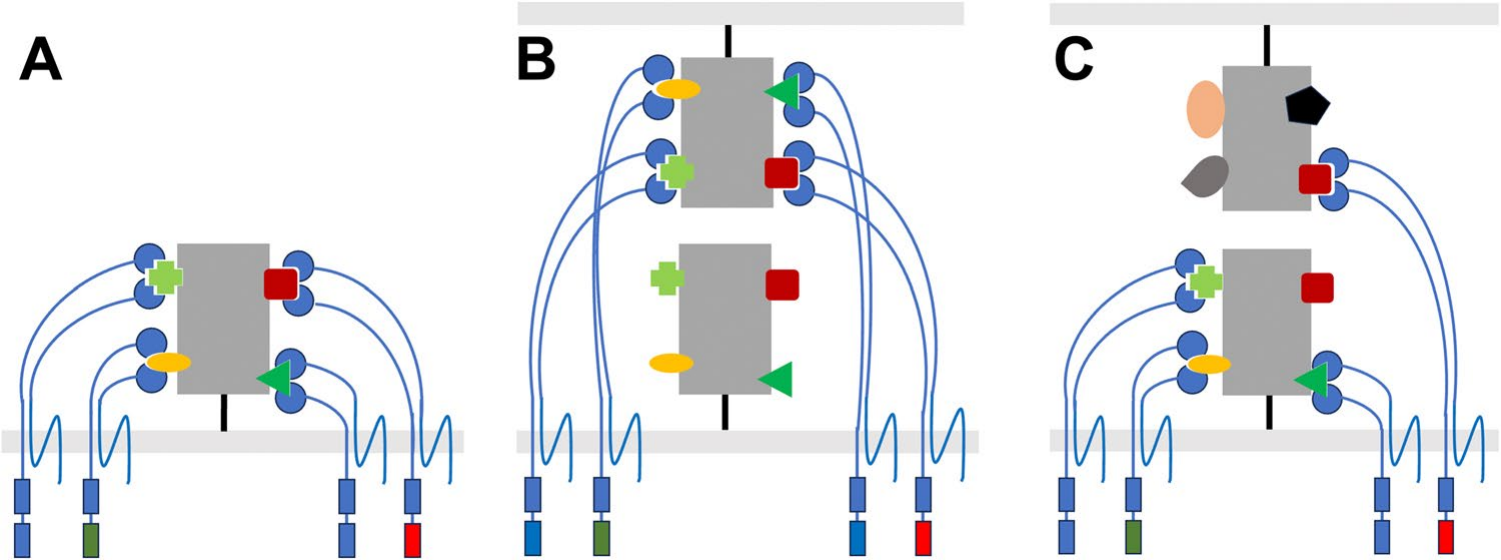
Working model of allorecognition specificity. **A** We hypothesize that under normal conditions, FF/FcoR heterodimers and fuhc-sec interact in cis. Shown are four different FF/FcoR heterodimers (blue) binding to four epitopes (outlined as colored shapes) on a single fuhc-sec allotype (gray box). The IgV regions of the FF/FcoR receptor heterodimers form a binding site for different ligand epitopes, and each FcoR encodes one or more tyrosine motifs (green = ITIM; blue = core motif; red = hemITAM). The cis interactions organize the signaling motifs into a structure that is required for allelic discrimination, affecting downstream competition between kinases and phosphatases. In turn, the FF/FcoR repertoire and signaling motifs are mixed and matched via an education process that also goes on in cis (described in text). **B**, **C** When ampullae from another individual comes into contact at the immune synapse, there is competition between cis and trans binding which will change the balance of signaling. If the two fuhc-sec allotypes match (as shown in **B**), the receptors from each individual switch form cis to trans binding (only one side shown here for clarity), causing coalescence and creation of a signaling platform that transmits a signal for fusion. **C** If only a few epitopes match (outlined as colored shapes), this would limit coalescence, in turn slowing inhibitory signaling and transmitting a signal initiating rejection

We envision that allelic discrimination in *Botryllus* is an extension of these processes. Specifically, each FF/FcoR pairing binds either polymorphic or conserved epitopes of *fuhc-sec*, providing a number of independent activating and inhibitory signals that are integrated—exactly as described for NK cells, but focused on a single ligand (Fig. 4). Two observations support this hypothesis: first, the fact that *uncle fester* is not ubiquitous means that our results using receptor crosslinking were genotype-specific, and there is not a single species level activating ligand or pathway as we had concluded previously (McKitrick et al. 2011). Second, the number of monomorphic FcoRs with hemITAMs and core motifs, but not ITIMs, as well as the two oligomorphic FcoRs that can switch between putative activating and inhibitory roles indicates that multiple activation signals are part of allelic discrimination. This also implies that *fuhc-sec* is both the activating and inhibitory ligand, consistent with the presence of a species level activation step (McKitrick et al 2011). This would require that *fuhc-sec* is at the PM, likely heterodimerizing with *fuhc-tm* or another membrane protein.

Our hypotheses on specificity are based on the fact that ampullae can be ablated and regenerate with no loss of specificity, and that genotype-specific expression levels and splice variants of both the FF and FcoR loci are maintained in the regenerated cells, even following multiple ablation/regeneration cycles (Nyholm et al. 2006; Taketa et al. 2024; Rodriguez-Valbuena et al. 2024). We propose that at the tips of individual ampullae, both activating and inhibitory FF/FcoR pairs bind epitopes of *fuhc-sec* in cis (Fig. 4A), and when two ampullae come together and form an immune synapse, this initiates competition between cis and trans binding, which would change the balance of ITAM/ITIM signals in both individuals (Fig. 4B, C). If the two f*uhc-sec* genes match at all epitopes, trans binding would initiate assembly into a higher order oligomeric structure that is required for strong inhibitory signal propagation and fusion (Boni et al. 2022). While the model in Fig. 4 shows four epitopes for clarity, population genetic studies of *fuhc-sec* reveal that more than 24 residues have strong evidence of positive selection (Fig. 3; Nydam et al. 2013c), suggesting a diversity of potential epitopes on each allotype.

In contrast, if epitopes are not completely shared, partial binding would initiate a rejection response (Fig. 4C), the intensity of which would be due to the number of activating and inhibitory epitopes which were shared between the two allotypes. We envision that there are a number of common epitopes shared between fuhc-sec allotypes that are species-specific and initiate rejection responses. This may explain why there are more monomorphic, non-ITIM FcoR loci. Finally, if no epitopes were shared, colonies would neither fuse nor reject, and this is consistent with studies on xenorecognition between botryllid species, all of which have a similar fuhc locus organization and express the same candidate proteins described here (Hirose et al. 2002; Rodriguez-Valbuena et al. in preparation). In summary, we speculate that fusion requires coalescence of a large receptor cluster, while rejection can be initiated from smaller clusters and is due to disruption of cis interactions.

As described above, we hypothesize that specificity is due to an education process that occurs during development. This includes genotype-specific expression and alternative splicing of the ectodomain of each FF and FcoR gene to allow the cell to match physical properties of the receptors to the allelic diversity of the self fuhc-sec epitopes, integrating multiple activating and inhibitory signals, and this would occur in cis (Fig. 4A). The cell can tune signaling by changing the activating and inhibitory functions of the oligomeric FF/FcoR receptor pairs by alternative splicing, as well as modifying other components of the signal transduction pathway, which could be the role of the polymorphic cytoplasmic proteins expressed in the fuhc-L: *HE3L*, *HSP40L*, and *BHF*. Similar to the rheostat model of NK cell education (Brodin et al. 2009), the cell somehow knows what the balance of inputs should be to set the correct activation threshold, with one major difference. Whereas education in mammalian NK cells balances signal strength with the stochastically expressed receptors to maintain tolerance, it does not affect the receptor repertoire itself, as manifested by the number of NK cells that are rendered hyporesponsive due to a lack of inhibitory ligands for self (Fernandez et al. 2005; Kim et al. 2005). In *Botryllus*, education would necessarily include feedback and modification of FF/FcoR expression, splicing and tuning—analogous to the tuning mechanisms that induce receptor editing during lymphocyte development (Nemazee 2006; Prak et al. 2011). This education process could either occur during embryogenesis and is epigenetically locked, similar to NK receptor repertoires (Santourlidis et al. 2002), or is a dynamic process that recurs in cis during regeneration. Both explain maintenance of specificity over time; however, we favor the latter, as one interpretation of the fester loss-of-function data is that tonic signaling is required to maintain alloreactivity (discussed below). However, the result in either case is a nanocluster consisting of both ligand and the FF/FcoR receptor repertoire interacting in cis (Fig. 4A).

If allelic discrimination depends on the degree of assembly of signaling competent higher order structures induced by trans binding, then the nanoscale organization of FcoR signal transduction motifs maintained by cis interactions with *fuhc-sec* is likely a critical component (Fig. 4A). This hypothesis is based on studies of receptor/ligand interactions where cis assembly of both the ligand and receptor has been shown to be critical for initiating receptor signaling following cell–cell contact. For example, structural studies on the interaction between the poliovirus receptor (PVR) and its inhibitory ligand TIGIT have revealed that self-assembly of TIGIT in cis is a critical component of PVR signaling following their interaction in trans. Specifically, it was found that TIGIT assembles in cis, but the homodimers are very weakly associated. When cells expressing two structure-guided TIGIT mutants that disrupted cis homodimerization were tested for binding to PVR-Fc in trans, each was equivalent to cells expressing wild-type TIGIT, demonstrating that these mutations had no effect on binding of the ectodomains. However, when the same control and mutant cell lines were paired with dendritic cells expressing PVR, tyrosine phosphorylation of PVR was significantly reduced when stimulated with cells expressing the mutated TIGIT vs those expressing wild-type TIGIT, as well as control stimulation with TIGIT-Fc (Stengel et al. 2012). In summary, despite the fact that the TIGIT and PVR ectodomains bound normally, and further that the TIGIT interactions in cis are very low affinity, PVR signaling following cell–cell contact was affected by the cis assembly of TIGIT, demonstrating that assembly on one side of the interaction affected trans-based oligomerization and signaling on the other, even though they are different binding interfaces. A role for cis assembly in trans binding strength has also been shown in studies on nectin-1, synCAM1, and both the cadherins and clustered protocadherins. In cadherins, cis and trans interactions are mutually cooperative, and in protocadherins, the coordination between cis and trans assembly is obligatory for binding and self/non-self recognition (Yap et al. 1997; Fogel et al. 2011; Narita et al. 2011; Thompson et al. 2021; Goodman et al. 2022).

If organization of the intracellular signal transduction platform can affect signaling, this is another place to discriminate between alleles. In T-cells, it has been shown that phosphorylation of CD3 ITAMs is dependent on TCR nanocluster density, which positively correlates with the affinity of the TCR/pMHC interaction (Pageon et al. 2016). A role of coalescence in allelic discrimination is also seen in the next step of TCR signaling, where it was found that the dwell time of an individual pMHC/TCR correlated with the probability of formation of a LAT condensate. Each phase transition is a quantum of information, as the number of LAT condensates correlates with the probability of NFAT translocation to the nucleus, and hence T-cell activation (McAffee et al. 2022). In addition, the spatial organization of activating and inhibitory motifs and their role in signal integration have been described in both T-cells (Yokosuka et al. 2012) and NK cells (Pageon et al. 2013). An analogous process could occur in *Botryllus*, as the proximity of hemITAM and ITIM and the other tyrosine core motifs of FF/FcoR pairs that are held in place by their interactions in cis could affect how initial phosphorylation events of those domains are controlled during binding in trans, and affect downstream interactions between kinases and phosphatases (Fig. 4A). This would explain why two of the FcoR loci would use alternative splicing to switch between activating and inhibitory function.

In addition, while the function of the tyrosine core motif on the FcoR proteins is not known, the residues upstream are equivalent to several of the phosphorylation sites on LAT and SLP-76; the two vertebrate signal transduction adaptors conspicuously absent in ascidians. This includes the presence of an acidic amino acid upstream of the tyrosine on half of them, a modification which has been shown to change the speed of phosphorylation, and plays a critical role in polymorphic discrimination by the TCR (Lo et al. 2023). If allelic discrimination is due to changes in oligomerization, some of the FcoR proteins may function as adaptors, and their positioning may be critical to proximal signal transduction. Our data also suggests this assembly may be dependent on BHF. We have found that when a fluorescently tagged version of *BHF* is co-expressed with *uncle fester* also expressing a cytoplasmic fluorescent tag, the two co-localize. The disordered nature of BHF could also be responsible for phase transitions that change the density upon trans interactions, and explain BHF loss-of-function results, which blocked both fusion and rejection responses (Voskoboynik et al. 2013) It is also intriguing to note that with the exception of a TM domain, the IDR regions of BHF are reminiscent of LAT, including the presence of several core tyrosine motifs (Y-x-x-I/L) equivalent to those of the FcoR, and targets of syk family phosphorylation (Lo et al. 2023).

Since the FF/FcoR are germline encoded, the specificity that can be generated will necessarily be diverse and genotype-specific—some individuals may have multiple FF/FcoR pairs that bind polymorphic epitopes tightly, others may have a several high affinity and several low affinity interactions, and some will only have lower affinity interactions. These differences would result in some genotypes having high binding specificity to one or both self *fuhc-sec* allotypes, while others produce intermediate, or low. Since education sets activation thresholds, those genotypes that can bind polymorphic epitopes strongly will necessarily have higher activating potential, likely reflected in changes in expression of monomorphic activating pairs such as *uncle fester*/*FcoR1*, and explain the phenotypic variation in rejection responses (described above; Taketa et al. 2024). This would be analogous to NK cells, where it has been shown that the activation potential positively correlates with the inhibitory inputs during education, due to the number of expressed inhibitory receptor/cognate ligand pairs (Joncker et al. 2009).

As described above, fusion can lead to stem cell parasitism, which equals death from an evolutionary perspective, so if a mistake is to be made, it is better to reject or not react to self, versus fusing with non-self. This suggests that education in *Botryllus* would set an activation threshold that is opposite that of NK cells—the potential for rejection would be slightly higher than fusion, which would require strong inhibitory signaling to overcome. From a spatial perspective, rejection could be initiated from scattered ligand/receptor clusters, similar to NK cells (Abeyweera et al. 2011), but fusion would require the coalescence and assembly of a larger structure. So how would genotype pairings with low specificity and thus low activation potential maintain specificity? As described above, mAb crosslinking studies of *fester* showed that both ampullae must be stimulated for fusion to occur, so each must discriminate self from non-self independently, no matter how weak the rejection barrier (Nyholm et al. 2006). Finally, what would the outcome be when two low affinity genotypes come together, each with little allelic discriminatory capabilities? They would likely be either non-reactive, or fuse and be potential casualties of stem cell parasitism. Some genotypes have to pay the price for fuhc diversity.

Finally, new specificities would arise as point mutations and intragenic combinations created new epitopes in *fuhc-sec* (Nydam et al. 2013c). As described above, the unique genomic redundancy of both FF and FcoR make these pairs highly evolvable, allowing them to adapt to these changes. Besides genomic diversification due to clustering, some of the pairs are linked to the fuhc-L, allowing maintenance of haplotype specific interactions. The presence of an unlinked haplotype allows the paired receptors to diversify, without affecting the other locus. In addition, the 3rd FcoR haplotype would allow for further diversification of that gene, and potentially experiment with and create new pairings, while others would become obsolete, resulting in the gene content variation of each haplotype. The creation of new epitopes and receptor interactions would be analogous to the evolutionary steps that resulted in the HLA-C2/KIR2DL1 interaction in the primates (Guethlein et al. 2015).

## Education and tonic signaling

For both T- and NK cells, effector specificity is not encoded in the genome, rather due to a cell autonomous quality control process during development that takes randomly generated binding specificities due to somatic recombination (TCR) or stochastic expression (NK receptors) and sets activation thresholds that allow a cell to be self-tolerant but functional, a process called education (Brodin et al. 2009; Joncker et al. 2009; Ashby and Hogquist 2024). Cells which do not pass this test are either ablated or rendered hyporesponsive, but how a T-cell knows the range between a positive and negative selection threshold, or NK cells balance activating and inhibitory inputs, is not understood.

In mammals, an equivalent education process is required for effector function in five unrelated innate receptor/ligand pairs: KIR-MHC Class 1; Ly49-MHC Class I; CD94/NKG2-HLA E (Qa-1); Nkrp1-Clr-b; and Sirpα-CD47. Each uses a missing-self strategy to determine the health of target cells, and in each knockout of the inhibitory ligand (MHC Class I; Clr-b; or CD47), which would predict an autoimmune phenotype, does exactly the opposite—resulting in tolerance (Liao et al. 1991; Vitale et al. 2002; Jaiswal et al. 2009; Chen et al. 2015). In addition, in NK cells, it has been demonstrated that this tolerance is reversible—NK cells that develop in an MHC deficient background are anergic, but regain functionality after being transplanted into a wild-type background, and wild-type NK cells can acquire tolerance when transplanted into an MHC deficient background (Elliott et al. 2010; Joncker et al. 2010). Similarly, we had found that knockout of the inhibitory receptor *fester* blocked both fusion and rejection responses from occurring. As discussed above, we had originally hypothesized that *fester* might be a subunit of both activating and inhibitory receptors (Nyholm et al. 2006). However, in retrospect, this could also be revealing an evolutionarily conserved characteristic of ITIM/ITAM signal integration—constant inhibitory signaling is required for maintenance of effector function in mature cells, and in *Botryllus*, this would occur in cis (Fig. 4). This dynamic tuning, or tonic signaling, is also required to maintain T-cell function in the periphery, suggesting that effector function in all these systems is dependent on a constant interaction with self-ligands. It is also interesting to note that in T-cells, NK cells, and most likely *Botryllus*, the rheostat concept is germane: the stronger the interaction with self, the higher the immune reactivity of the cells (Hogquist and Jameson 2014; Brodin et al. 2009). While in NK cells and *Botryllus* the interaction with self is via inhibitory receptors, in T-cells, it is due to the TCR quantitating binding to self pMHC (Azzam et al. 1998). Consequently, a single TCR can set an activation threshold by itself, a process that requires two receptors in a NK cell. In that context, the TCR could also be thought of as using a missing-self recognition strategy.

## Conclusions

Studies of histocompatibility in both *Botryllus* and *Hydractinia* indicate that the mechanisms that underlie polymorphic discrimination rely on the dynamic range provided by the interaction between ITAM and ITIM signaling pathways, and had an early evolutionary origin. Both NK and T-cells assess the health of a target cell by detecting changes in self-ligands. To make that comparison, both cells must be “educated” to what normal self is during development, and both require constant input from self in the periphery to maintain tolerance. The conservation of the mechanisms which allows cells to compare two states also permits the receptor ectodomains to evolve freely, and explains the rapid, recurrent, and convergent evolution of missing-self recognition systems throughout the vertebrates (Kelley et al. 2005; Carlyle et al. 2008; Guethlein et al. 2015), as well as the emergence of the cellular branch of adaptive immunity (Flajnik and Kasahara 2010).

For the latter, it has been hypothesized that the critical moment in the evolution of adaptive immunity was the invasion of an antigen receptor by the RAG transposon in a precursor to the jawed vertebrates, which initiated the diversification of antigen receptors that is the basis of anticipatory immunity (Flajnik and Kasahara 2010). For the TCR, creating immune diversity using random somatic recombination requires both positive and negative selection. How could a transposon insertion initiate the evolution of a system that has a 98% failure rate, and require the evolution of the thymus, if immune cells did not already know how take diversity and create specificity, and hence tolerance? We and others have hypothesized that RAG had to have invaded a missing-self receptor that was already involved in polymorphic discrimination (Pasquier 2000; Flajnik and Kasahara 2001). This could have been the same interaction as occurs today with NK cells—downregulation of a health ligand, such as MHC Class I or clr-b, is a sign of infection, and the inhibitory receptors used to detect stress would be competing with pathogen decoy molecules to maintain specificity (Orange et al. 2002; Aguilar et al. 2017). This is a strong selective pressure that would result in the evolution of a polymorphic recognition strategy (Carlyle et al. 2008). It is also possible that an allorecognition system analogous to those in *Botryllus* or *Hydractinia* was present in the placoderm lineage, and a receptor in that region was targeted. In either case, polymorphic discrimination is dependent on cell autonomous education processes that likely existed long before the emergence of vertebrates, and would have been co-opted for thymic selection following the acquisition of somatic diversification. The unique characteristics of *Botryllus* allorecognition make it an excellent model to dissect the fundamental mechanisms which underly specificity.
